# Supplementary material for: Antioxidant and Nitric Oxide Inhibitory Activity of the Six Most Popular Instant Thai Curries
Source: Foods. 2024 Jan 5;13(2):178. doi: 10.3390/foods13020178 (PMC10814089; doi:10.3390/foods13020178)
Supplement: Supplementary file 1 [file foods-13-00178-s001.zip › foods-2745146-supplementary.pdf]

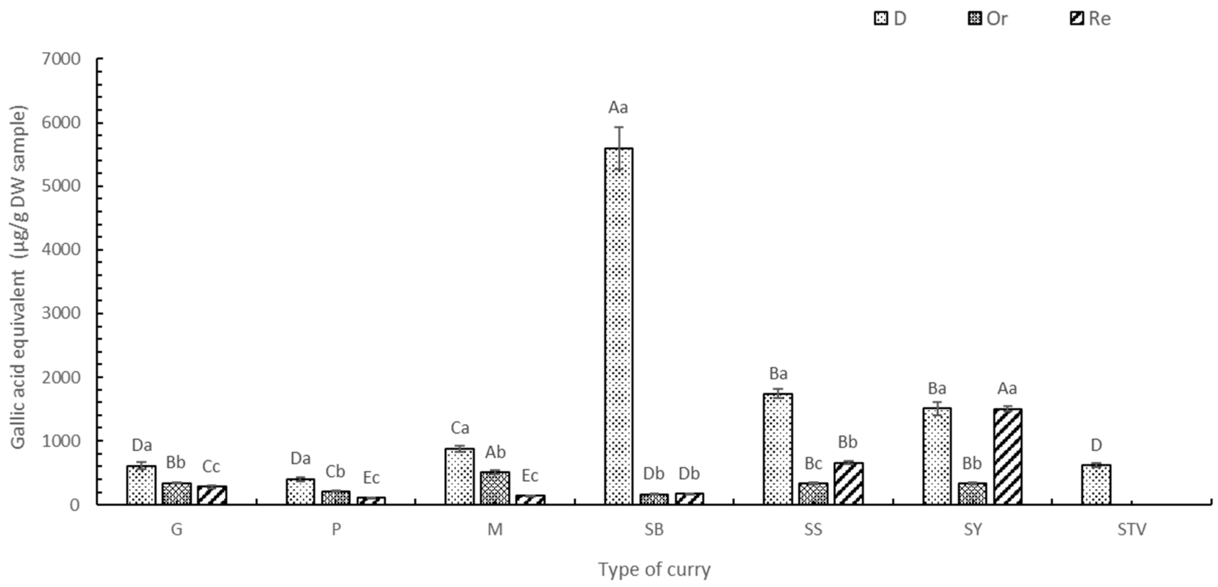

Figure S1 TPC of curry and stevia extracts using gallic acid as a standard.

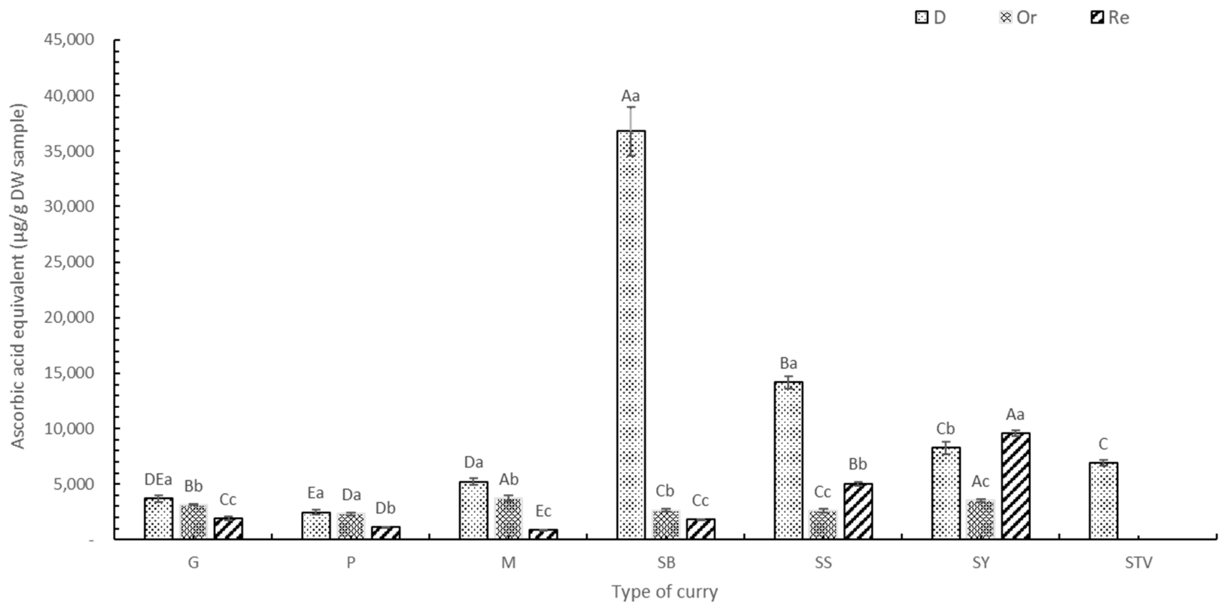

Figure S2 TPC of curry and stevia extracts using ascorbic acid as a standard.

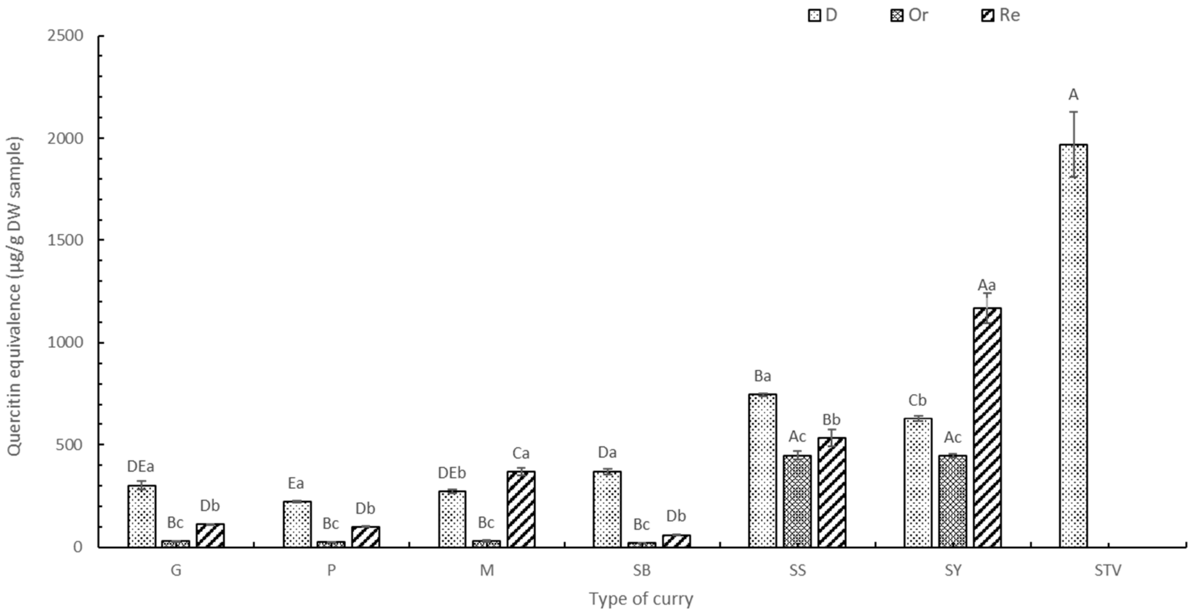

Figure S3 TFC of curry and stevia extracts using quercetin as a standard.

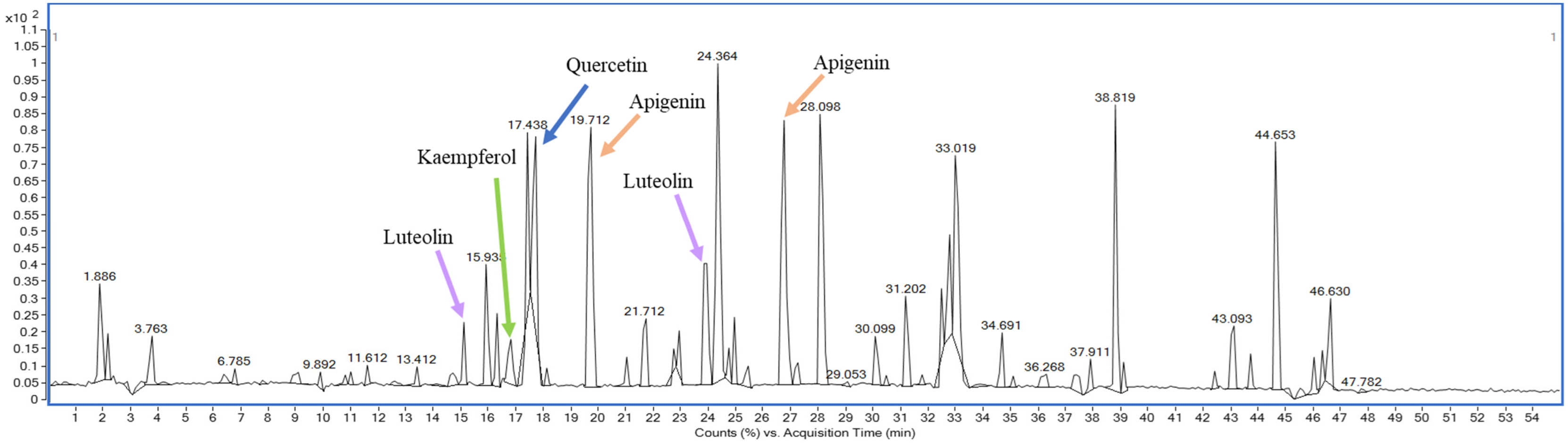

Figure S4 Chromatogram of phenolic compounds found in green curry (G) deter-mined by LC-ESI-QTOF-MS/MS.

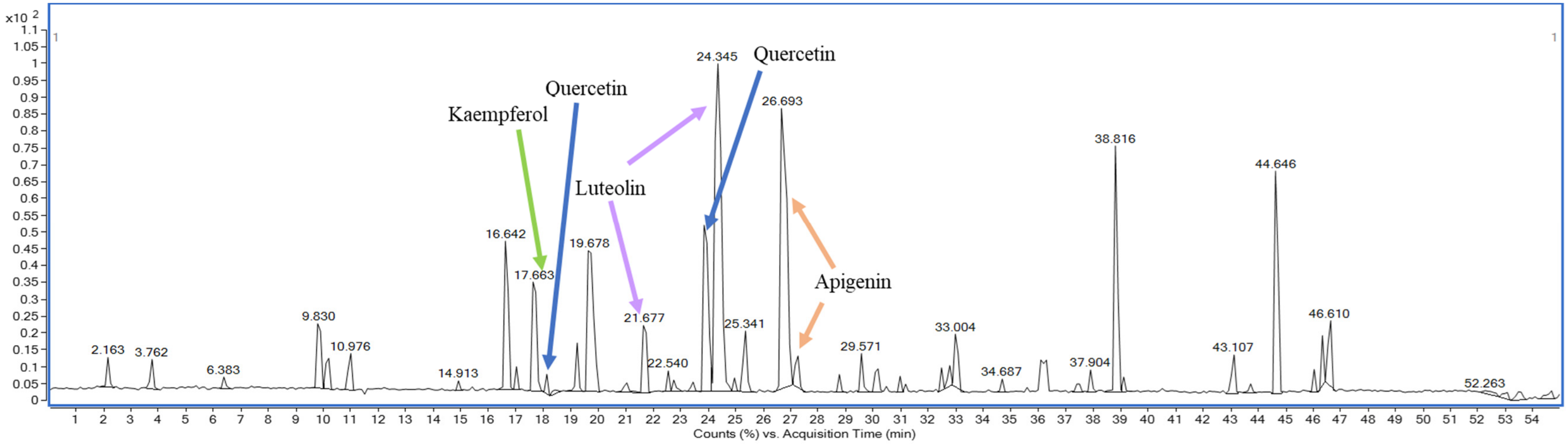

Figure S5 Chromatogram of phenolic compounds found in Panang curry (P) deter-mined by LC-ESI-QTOF-MS/MS.

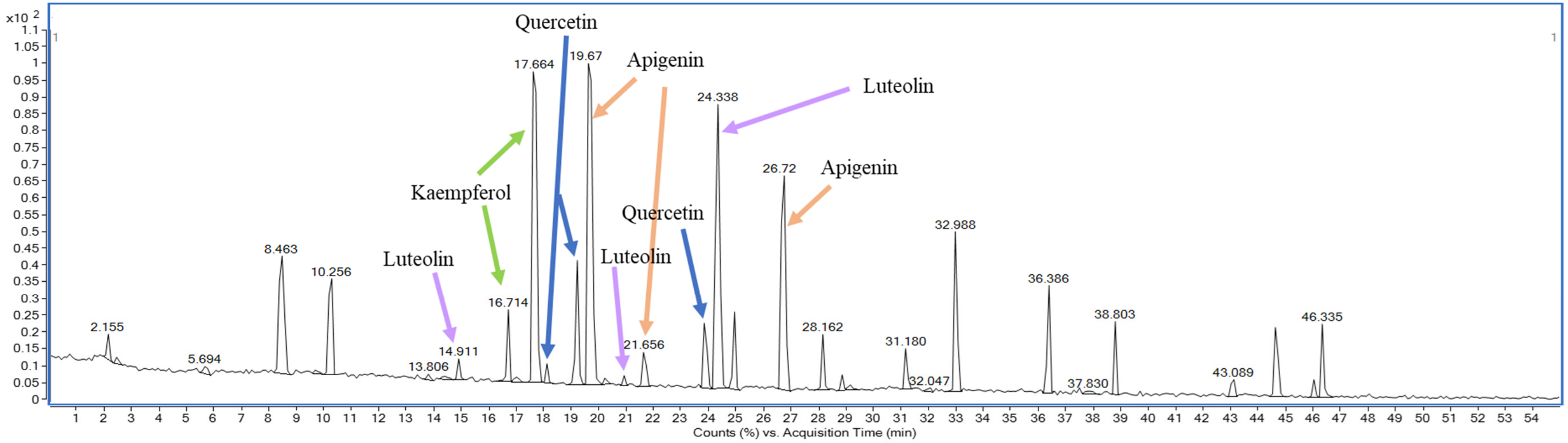

Figure S6 Chromatogram of phenolic compounds found in Massaman curry (M) deter-mined by LC-ESI-QTOF-MS/MS.

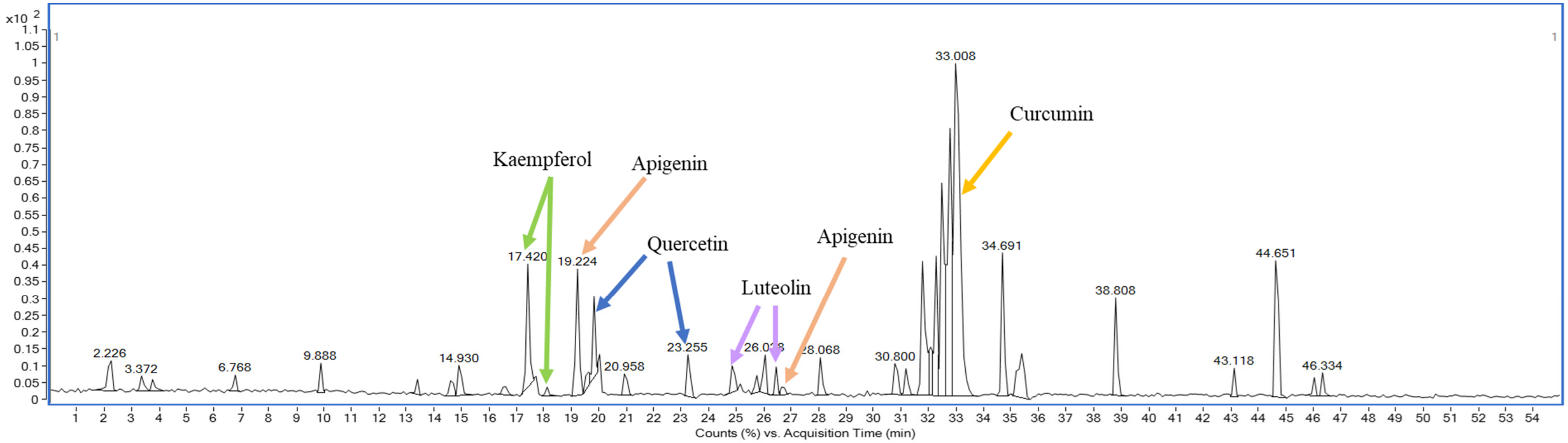

Figure S7 Chromatogram of phenolic compounds found in southern sour curry (SS) deter-mined by LC-ESI-QTOF-MS/MS.

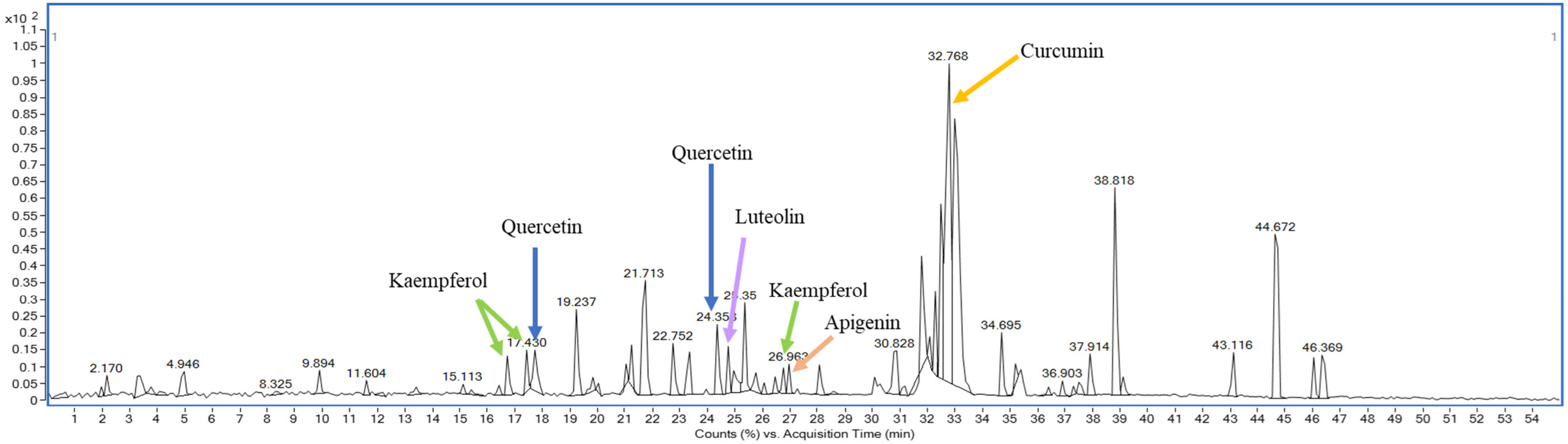

Figure S8 Chromatogram of phenolic compounds found in southern spicy yellow curry (M) deter-mined by LC-ESI-QTOF-MS/MS.

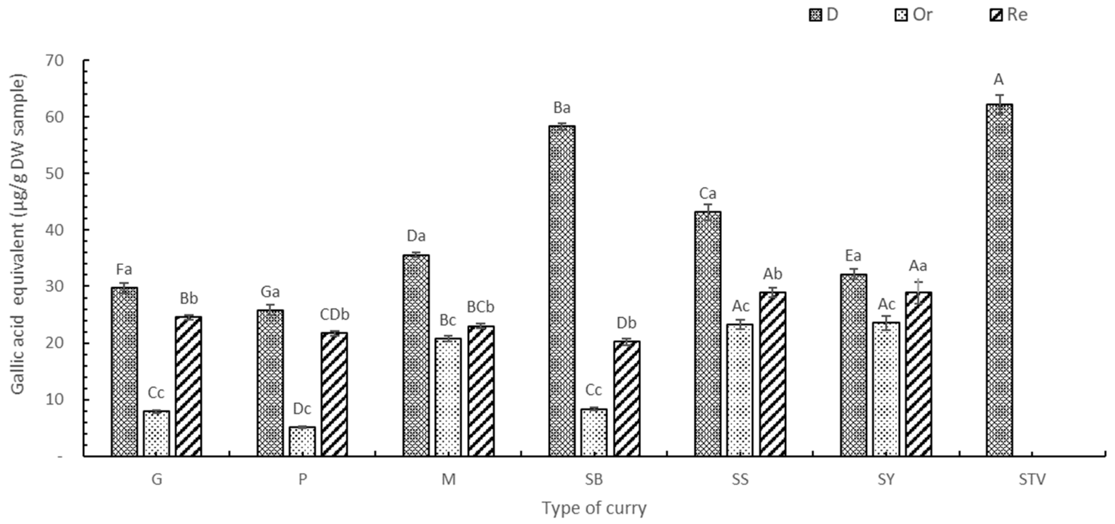

Figure S9 DPPH of curry and stevia extracts using gallic acid as a standard.

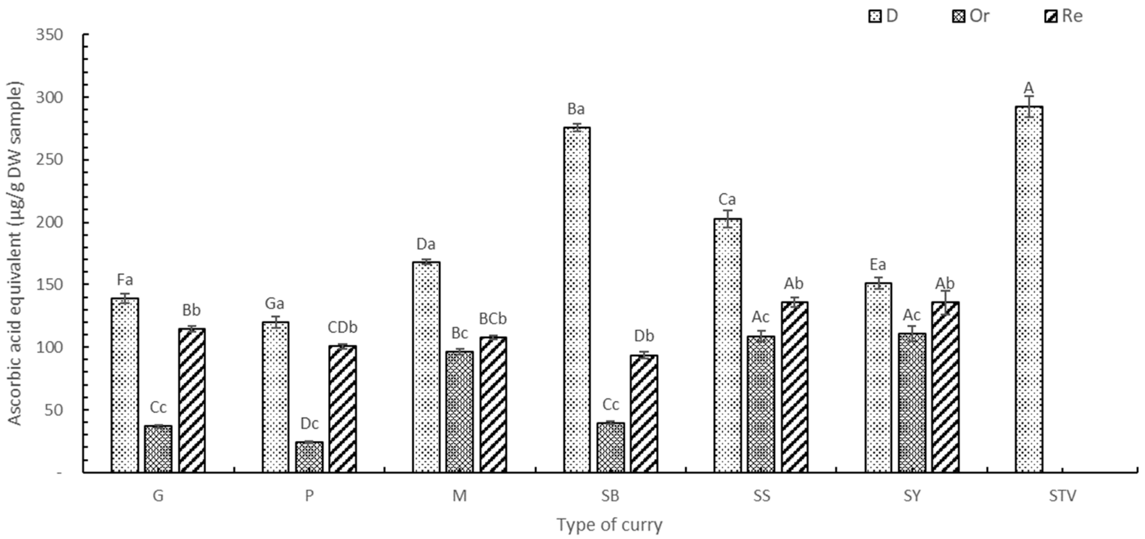

Figure S10 DPPH of curry and stevia using ascorbic acid as a standard.

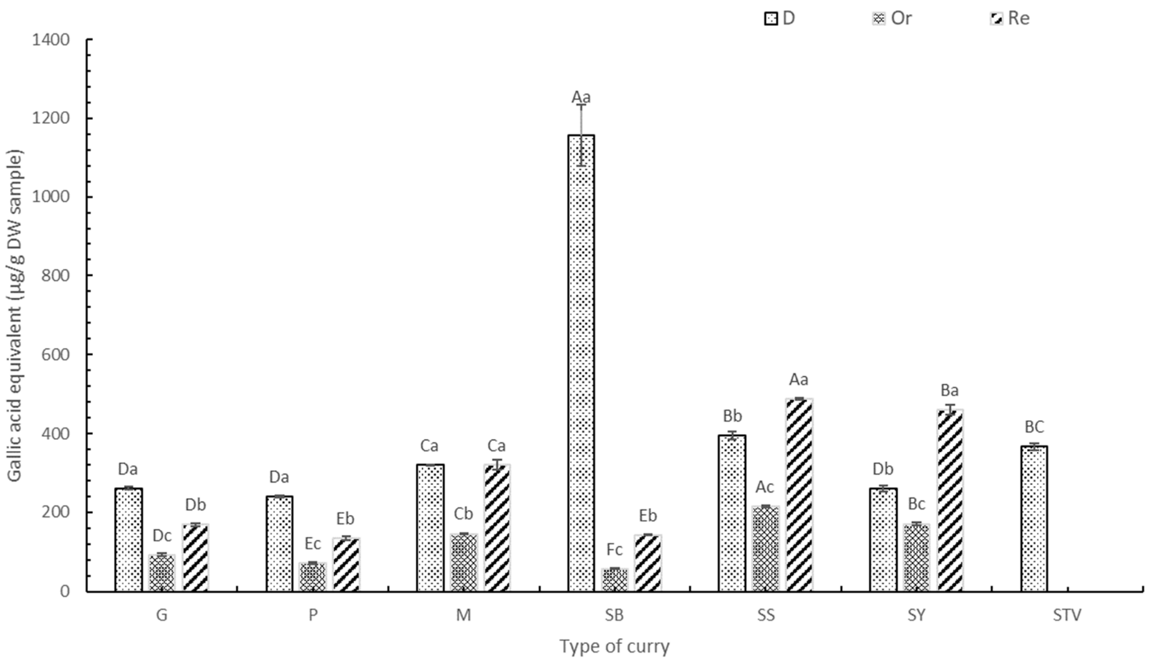

Figure S11 ABTS of curry and stevia extracts using gallic acid as a standard.

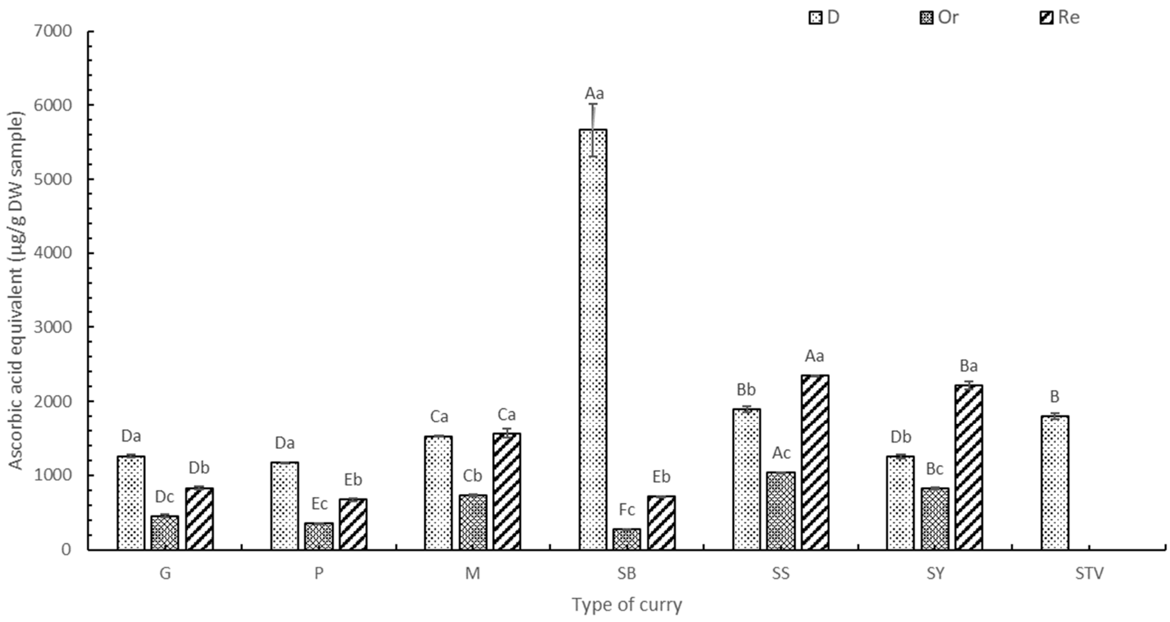

Figure S12 ABTS of curry and stevia extracts using ascorbic acid as a standard.

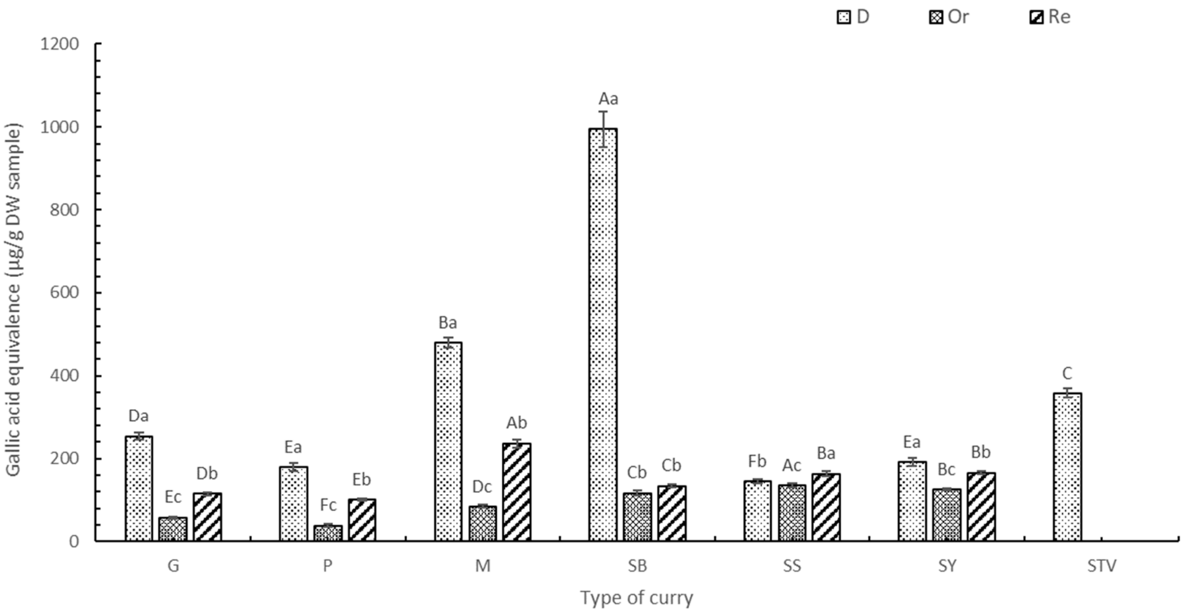

Figure S13 FRAP of curry and stevia extracts using gallic acid as a standard.

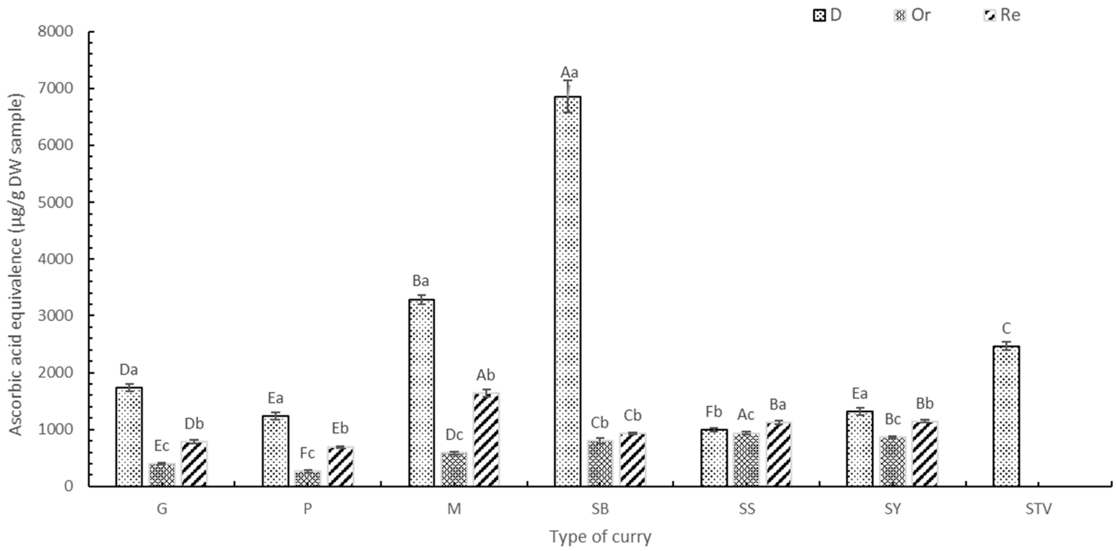

Figure S14 FRAP of curry and stevia extracts using ascorbic acid as a standard.

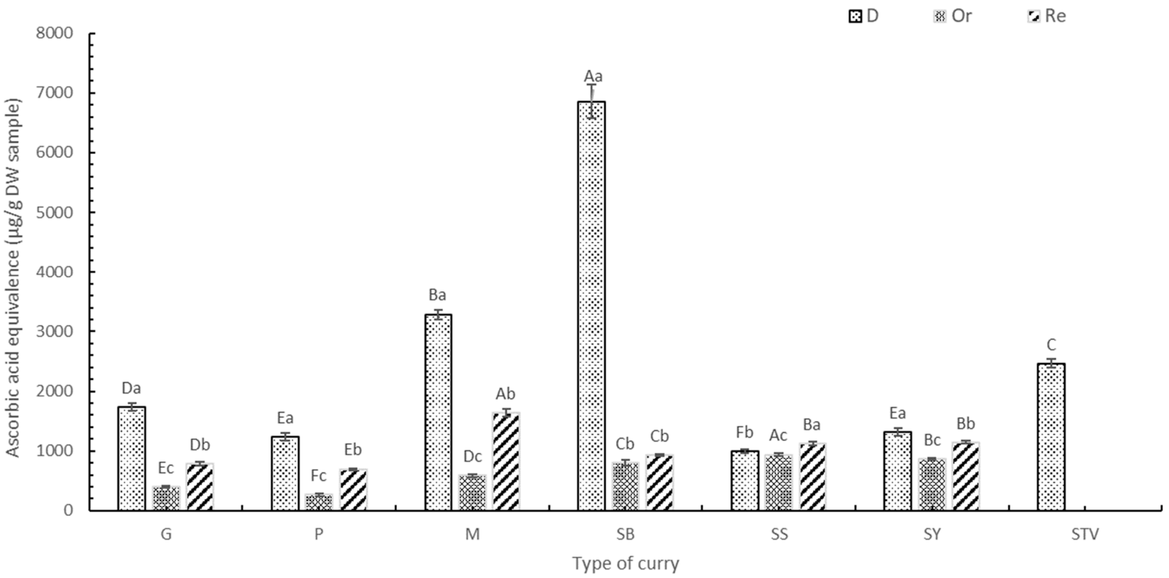

Figure S15 FRAP of curry and stevia extracts using $FeSO_4$ as a standard.
